# Early pre-radiographic structural pathology precedes the onset of accelerated knee osteoarthritis

**DOI:** 10.1186/s12891-019-2624-y

**Published:** 2019-05-22

**Authors:** Matthew S. Harkey, Julie E. Davis, Bing Lu, Lori Lyn Price, Robert J. Ward, James W. MacKay, Charles B. Eaton, Grace H. Lo, Mary F. Barbe, Ming Zhang, Jincheng Pang, Alina C. Stout, Timothy E. McAlindon, Jeffrey B. Driban

**Affiliations:** 1 Division of Rheumatology, Allergy, and Immunology, Tufts Medical Center, 800 Washington Street, Box 406, Boston, MA 02111 USA; 2 Department of Population and Quantitative Health Sciences, University of Massachusetts Medical School, Worcester, MA USA; 3 Division of Rheumatology, Immunology & Allergy, Brigham & Women’s Hospital and Harvard Medical School, Boston, MA USA; 4 The Institute for Clinical Research and Health Policy Studies, Tufts Medical Center, Boston, MA USA; 5 Tufts Clinical and Translational Science Institute, Tufts University, Boston, MA USA; 6 Department of Radiology, Tufts Medical Center, Boston, MA USA; 7 Department of Radiology, University of Cambridge School of Clinical Medicine, Cambridge, UK; 8 Center for Primary Care and Prevention, Alpert Medical School of Brown University, Pawtucket, RI USA; 9 Medical Care Line and Research Care Line, Houston Health Services Research and Development (HSR&D) Center of Excellence Michael E. DeBakey VAMC, Houston, TX USA; 10 Section of Immunology, Allergy, and Rheumatology, Baylor College of Medicine, Houston, TX USA; 11 Department of Anatomy and Cell Biology, Temple University School of Medicine, Philadelphia, PA USA; 12 Department of Computer Science & Networking, Wentworth Institute of Technology, Boston, MA USA; 13 Pfizer Inc., Cambridge, MA USA; 14 Public Health Institute, Northeastern University, Boston, MA USA

**Keywords:** Cartilage, Bone marrow lesion, Effusion-synovitis, Meniscus, Ligament, Tendons, Extensor mechanism, MRI

## Abstract

**Background:**

Accelerated knee osteoarthritis (AKOA) is characterized by more pain, impaired physical function, and greater likelihood to receive a joint replacement compared to individuals who develop the typical gradual onset of disease. Prognostic tools are needed to determine which structural pathologies precede the development of AKOA compared to individuals without AKOA. Therefore, the purpose of this manuscript was to determine which pre-radiographic structural features precede the development of AKOA.

**Methods:**

The sample comprised participants in the Osteoarthritis Initiative (OAI) who had at least one radiographically normal knee at baseline (Kellgren-Lawrence [KL] grade < 1). Participants were classified into 2 groups based on radiographic progression from baseline to 48 months: AKOA (KL grade change from < 1 to > 3) and No AKOA. The index visit was the study visit when participants met criteria for AKOA or a matched timepoint for those who did not develop AKOA. Magnetic resonance (MR) images were assessed for 12 structural features at the OAI baseline, and 1 and 2 years prior to the index visit. Separate logistic regression models (i.e. OAI baseline, 1 and 2 years prior) were used to determine which pre-radiographic structural features were more likely to antedate the development of AKOA compared to individuals not developing AKOA.

**Results:**

At the OAI baseline visit, degenerative cruciate ligaments (Odds Ratio [OR] = 2.2, 95% Confidence Interval [CI] = 1.3,3.5), infrapatellar fat pad signal intensity alteration (OR = 2.0, 95%CI = 1.2,3.2), medial/lateral meniscal pathology (OR = 2.1/2.4, 95%CI = 1.3,3.4/1.5,3.8), and greater quantitative knee effusion-synovitis (OR = 2.2, 95%CI = 1.4,3.4) were more likely to antedate the development of AKOA when compared to those that did not develop AKOA. These results were similar at one and two years prior to disease onset. Additionally, medial meniscus extrusion at one year prior to disease onset (OR = 3.5, 95%CI = 2.1,6.0) increased the likelihood of developing AKOA.

**Conclusions:**

Early ligamentous degeneration, effusion/synovitis, and meniscal pathology precede the onset of AKOA and may be prognostic biomarkers.

## Background

While knee osteoarthritis (OA) is typically is a gradually progressive disorder, a subset of individuals develop an accelerated form of the disease that is defined by the rapid onset and progression of disease within 4 years, and oftentimes within 12 months [1–3]. Accelerated knee OA (AKOA) represents a greater personal burden compared to typical knee OA, because individuals with AKOA are more likely to report frequent knee pain and greater self-reported global impact of arthritis (i.e., 0–10 global rating scale), as well as present with decreased physical function performance (e.g. slower walking and chair-stand pace) [2]. Additionally, AKOA represents an increased economic burden, as individuals with AKOA are more likely to receive pharmacological/surgical treatments and knee replacements compared with individuals with typical knee OA [4]. Therefore, developing prognostic tools that can distinguish between people who will develop AKOA are needed to lessen the personal and economic burden of this disease.

There is preliminary evidence that alterations in the meniscus and subchondral bone may characterize the onset of AKOA [5]. However, since knee OA is a disease that affects all structures of the joint [6], a more in-depth investigation of alterations in pre-radiographic structural features is needed. Magnetic resonance (MR) imaging offers a comprehensive assessment that evaluates cartilage, subchondral bone, meniscus, ligaments, tendons, and synovium. The annual MR image assessments of the Osteoarthritis Initiative (OAI) in individuals with radiographically normal knees allows for the unique ability to monitor early pre-radiographic structural alterations prior to the rapid decline in joint health associated with AKOA.

The primary purpose of this analysis was to determine which pre-radiographic structural features at key OAI visits precede the radiographic development of AKOA. Due to the rapid radiographic decline in joint health in individuals with AKOA, we hypothesize that early degenerative changes in the cruciate ligaments, extensor mechanism, and proximal gastrocnemius tendons would be associated with the future onset of AKOA. Furthermore, we hypothesized the effusion/synovitis and the presence of meniscal pathology would be associated with the onset of AKOA. Additionally, we explored which combination of pre-radiographic structural features may best discriminate which individuals will develop AKOA. The results of these analyses will indicate which pre-radiographic structural features may be ideal prognostic imaging markers for future AKOA development. These imaging markers will be imperative for selecting individuals at risk for AKOA to assess and deploy prevention strategies for incident AKOA.

## Methods

### Study design and participant selection

We identified individuals for this study using radiographic data from the OAI baseline and first 4 annual follow-up visits. The OAI is a multicenter (Memorial Hospital of Rhode Island, The Ohio State University, University of Maryland and Johns Hopkins University, and the University of Pittsburgh) cohort study that recruited 4796 adults with or at risk for symptomatic knee OA between February 2004 and May 2006 [7]. Institutional review boards at all OAI clinical sites and the OAI coordinating center (University of California, San Francisco) approved the OAI study. Participants provided informed consent prior to participation.

For this study, readers assessed 12 knee features on MR images at the OAI baseline visit, as well as at specific timepoints relative to the onset of disease (i.e. 2 and 1 years prior to onset of disease). Key features include semi-quantitative readings (i.e. collateral ligaments, cruciate ligaments, extensor mechanism, gastrocnemius tendons, infrapatellar fat pad signal intensity alteration, menisci) and quantitative measures (i.e. effusion-synovitis, bone marrow lesion [BML], and cartilage).

### Participant selection

Participants in all groups were identified based on annual radiographs from the baseline to the 48-month OAI visit [3]. All groups had at least one knee with no radiographic knee OA at baseline (Kellgren-Lawrence [KL] < 1). Individuals who developed AKOA were defined as having one knee progress to advanced-stage knee OA (KL Grade = 0/1 to 3/4, definitive osteophyte and joint space narrowing) within 48 months (*n* = 125) [3]. Individuals with typical knee OA experienced a more gradual onset of OA and were defined as having one knee increase in KL grade within 48 months (i.e. KL = 0 to 1, 0 to 2, 1 to 2; *n* = 187). Individuals were defined as having no knee OA if both knees had no change in KL grade from baseline to the 48-month OAI visit (*n* = 1325). Individuals in the typical and no knee OA group were randomly matched to the AKOA group based on sex. Each group had 125 participants. For data analysis, we combined the typical knee OA and no knee OA groups into a single “no AKOA” group to allow for a comparison between individuals who would and would not develop AKOA [8].

### Index knee

The index knee in individuals with AKOA or typical knee OA was defined as the first knee to meet the definition of AKOA or typical knee OA, respectively. The index knee in individuals with no knee OA was the same knee as that person’s matched member of the AKOA group.

### Index visit

For individuals with AKOA or typical knee OA, the index visit was defined as the visit when a person first met the definition for AKOA or typical knee OA. For someone with no knee OA the index visit was the same visit as that person’s matched member of the incident AKOA group. The index visit could be at a 12-, 24-, 36-, or 48-month OAI visit.

### Knee radiographs

To determine group assignment, we used readings of bilateral weight-bearing, fixed-flexion posteroanterior knee radiographs obtained at baseline and each annual follow-up visits [3]. Central readers blinded to group assignment scored the KL Grade of each knee (KL = 0 to 4). The intrarater reliability agreement for the KL grades was good (weighted κ = 0.70 to 0.80) [9]. These data are publicly available (files: kXR_SQ_BU##_SAS [versions 0.6, 1.6, 3.5, 5.5, and 6.3]) [10].

### MR imaging

#### MR acquisition

All semi-quantitative and quantitative analyses were conducted in index knees at the OAI baseline visit, as well as at 2 and 1 years prior to the index visit. MR images were acquired with one of four identical Siemens (Erlangen, Germany) Trio 3-Tesla MR systems at each clinical site using the OAI MR imaging protocol [10, 11]. The two musculoskeletal radiologists (RW, JM) performing semi-quantitative scoring were provided all the sequences acquired on each index knee at each visit (e.g., sagittal intermediate-weighted, turbo spin echo, fat-suppressed MR sequence; coronal intermediate-weighted, turbo spine echo, sequence without fat suppression, 3-dimensional dual-echo steady-state sequence). The quantitative measures of BML and effusion-synovitis were performed using a sagittal intermediate-weighted, turbo spin echo, fat-suppressed MR sequence: field of view = 160 mm, slice thickness = 3 mm, skip = 0 mm, flip angle = 180 degrees, echo time = 30 ms, recovery time = 3200 ms, 313 × 448 matrix, *x* resolution = 0.357 mm, *y* resolution = 0.511 mm, and total slice number = 37. Cartilage damage index was quantified using a 3-dimensional dual-echo steady-state sequence: field of view = 140 mm, slice thickness = 0.7 mm, skip = 0 mm, flip angle = 25 degrees, echo time = 4.7 ms, recovery time = 16.3 ms, 307 × 384 matrix, *x* resolution = 0.365 mm, *y* resolution = 0.456 mm, and total slice number = 160. These sequences have been described in detail elsewhere [10].

#### Semi-quantitative structural features

For all semi-quantitative and quantitative outcomes, the readers were blinded to group assignment and were unblinded to the order of time. Two musculoskeletal radiologists (RW:255 cases, JM:120 cases) performed the semi-quantitative MR readings. Readers had good agreement on the presence of each pathology among 25 cases: prevalence-adjusted and bias-adjusted kappa were 0.41 to 0.75 except for the posterior horn of the medial meniscus where the prevalence-adjusted and bias-adjusted kappa was fair at 0.25 (50% agreement).

The radiologists assessed the integrity of anterior/posterior cruciate ligaments, medial/lateral collateral ligaments, extensor mechanism, and gastrocnemius proximal tendons and noted if the structures appeared normal or degenerative. Degenerative tissue was defined as the presence of abnormal intrinsic high-signal intensity within the substance of the ligaments or tendon without discrete tear. Degenerative cruciate ligament pathology combined the presence of anterior or posterior cruciate ligament degenerative pathology. Degenerative collateral ligament pathology combined the presence of medial or lateral collateral ligament degenerative pathology.

The radiologists scored infrapatellar fat pad signal intensity alteration using the MR Imaging Osteoarthritis Knee Score grading system (i.e., normal, mild, moderate, and severe) [12]. Infrapatellar fat pad signal intensity was recoded as absence (i.e., normal) or presence (i.e., mild, moderate, and severe).

The radiologists scored medial and lateral meniscus extrusion using the MR Imaging Osteoarthritis Knee Score grading system (i.e., Grade 0: < 2 mm, Grade 1: 2 to 2.9 mm, Grade 2: 3 to 5 mm, and Grade 3: > 5 mm) [12]. Meniscal extrusion was recoded as absence (i.e., Grade 0) or presence (i.e., > Grade 1).

The radiologists used the International Society of Arthroscopy, Knee Surgery, and Orthopaedic Sports Medicine meniscal tear classification, which was modified for MR imaging [13], to assess the body, posterior/anterior horn of each meniscus as: normal, degeneration, horizontal, flap horizontal, vertical longitudinal, radial, morphologic deformity, maceration, complex, or vertical flap tear. Meniscal pathology was recoded as absence (i.e., normal or degeneration without tear) and presence (i.e., horizontal, flap horizontal, vertical longitudinal, radial, morphologic deformity, maceration, complex, or vertical flap tear). For the medial/lateral menisci, pathology in the three regions meniscal tears of different morphologies were combined into the same variable. The medial/lateral menisci were considered pathologic if pathology was present in any of the three regions.

#### Quantitative structural features

##### Effusion-synovitis volume

We used a customized semi-automatic software to measure knee effusion-synovitis. Two readers (JBD and a visiting fellow) used the software to mark the first and last MR slice that included bone, the proximal border of the patella, and the apex of the fibular head on a central slice. The software then automatically segmented effusion-synovitis between these limits based on an existing threshold. The senior reader (JBD) then manually adjusted the threshold to change the effusion-synovitis boundaries and removed areas of high signal intensity that were not effusion-synovitis (e.g., subchondral cysts, blood vessels). The senior reader demonstrated excellent intra-reader reliability (ICC_3,1_ = 0.96). A total knee effusion-synovitis volume (in cm^3^) was used for data analysis.

##### Bone marrow lesion volume

One reader (ACS) measured tibiofemoral BML volume with a semi-automated segmentation method [14, 15]. The only manual step required the reader to identify crude boundaries of the tibia and femur in each slice of the MR images. The boundary furthest from the articular surfaces was marked just prior to the epiphyseal line or at the edge of the bone and soft tissue. The program then automatically identified the precise bone boundaries and performed a thresholding and curve evolution process twice to segment areas of high signal intensity, which may represent a BML. We eliminated false-positive regions by operationally defining a BML based on 2 criteria: 1) the distance between a BML to the articular surface should be < 10 mm; 2) a BML needed to span more than one MR image. The study principal investigator (JBD) reviewed all measurements with both timepoints on screen simultaneously. Our reader demonstrated excellent intra-reader reliability (ICC_3,1_ = 0.91). A total tibiofemoral BML volume (in cm^3^) was used for data analysis.

##### Cartilage damage index

The validated cartilage damage index (CDI) was used to quantify tibiofemoral cartilage size [16, 17]. One reader (JED) manually marked the bone-cartilage boundary on specific knee slices that are automatically selected based on the width of the knee. The reader then measured cartilage thickness at predefined informative locations, which the software automatically located. The software then computed the CDI for the medial femur, lateral femur, medial tibia, and lateral tibia by summing the products of cartilage thickness, cartilage length (anterior-posterior), and voxel size from 9 informative locations in each compartment. All measurements were reviewed by study principal investigator. Our reader demonstrated excellent intra-reader reliability (ICC_3,1_ = 0.86 to 0.99). The sum of all four tibiofemoral compartment CDI values was divided by the participant’s height to calculate a normalized total tibiofemoral CDI that was used for data analysis.

### Clinical data

Demographic and other participant characteristics were acquired based on a standard protocol. We extracted age, body mass index, global impact rating, frequent knee pain and Western Ontario and McMaster Universities Osteoarthritis Index (WOMAC) pain at the OAI baseline visit. The data are publicly available (Files: allclinical0#; version 0.2.2, 1.2.1, 3.2.1, 5.2.1, and 6.2.1) [11].

### Data analysis

For the continuous quantitative outcomes, the variables in the entire cohort were separated into tertiles and converted to a dichotomous variable to compare the worst tertile (i.e. largest BML and effusion-synovitis, smallest CDI) to the combination of the other two tertiles to facilitate the interpretation of the odds ratios.

### Statistical analysis

#### Primary analysis

*Are Early Pre-Radiographic Structural Features Associated with the Onset of Accelerated Knee Osteoarthritis?*

Separate logistic regression models were used to determine which pre-radiographic structural features at OAI baseline were more likely to antedate the development of AKOA compared to individuals not developing AKOA (i.e. referent group). Additionally, we conducted the same analyses for each structural outcome at 2 and 1 years prior to the index visit. The results are presented as odds ratios (ORs) and 95% confidence intervals (95% CIs). To control for multiple comparisons, we utilized a statistically significant *p*-value corrected for the number of structural features utilized in the primary OAI baseline analysis (*p* < 0.05/12 = 0.004).

#### Secondary analysis

*Which Combination of Baseline Pre-Radiographic Structural Features Most Associate with the Onset of Accelerated Knee Osteoarthritis?*

To explore which combination of pre-radiographic structural features characterize AKOA, we performed a backward stepwise logistic regression where the outcome was AKOA or no AKOA (referent group) at the OAI baseline. Separate models also were conducted for 2 and 1 years prior to the index visit. All 9 semi-quantitative and 3 quantitative pre-radiographic structural features were included in the analysis at each time point. The ability for the combination of pre-radiographic structural features to discriminate between AKOA status was quantified with the C statistic [18]. The discriminatory ability of a model based on the C statistic was classified as: very poor (C < 0.50), poor (0.50 < C < 0.70), good (0.70 < C < 0.80), and strong (0.80 < C < 1.00) [19].

All analyses were performed unadjusted, as the purpose of this investigation was to specifically determine the prognostic capability of baseline structural features at associating with future development of incident AKOA. Due to missing MR images at different OAI visits, there are different sample sizes depending on the analysis: OAI baseline (*n* = 354), 2 years prior to onset (*n* = 248), 1 years prior to onset (n = 354). There are uneven sample sizes at the different time points because some participants are unable to have a 2 year prior to onset visit (i.e., index visit at the 1-year OAI visit). We conducted a sensitivity analysis for the OAI baseline and 1 year prior to onset analyses limiting the sample to the 248 participants in the 2 years prior to onset analysis. All analyses were performed with SAS Enterprise 7.15 (Cary, NC, USA).

## Results

Table 1 details the demographics for each group.

**Table 1:** Baseline Descriptive Characteristics of Individuals with and without Incident Accelerated Knee Osteoarthritis (AKOA)

Variables	AKOA	No AKOA
[(means (SD); except where noted)	(*n* = 113)	(*n* = 241)
Females, n(%)	71(63%)	151(63%)
Index knee KL Grade = 0, n(%)^a^	37(33%)	157(65%)
Frequent knee pain in past 12 months, n(%)	62(57%)	75(31%)
Age (years)	65.6(8.4)	60.1(8.4)
Body mass index (kg/m^2^)	29.5(4.4)	27.5(4.5)
Global impact rating (0 to 10; ↑score = ↑ impact)	2.2(2.1)	1.0(1.4)
WOMAC pain (0 to 20; ↑score = ↑pain)	3.4(3.7)	1.6(2.6)

^a^Index knee Kellgren-Lawrence (KL) grade could be 0 or 1. *SD* standard deviation, *WOMAC* Western Ontario and McMaster Universities Osteoarthritis Index, *kg/m*^*2*^ kilogram per meters squared

### Primary analysis

*Are Pre-Radiographic Structural Features Associated with the Onset of Accelerated Knee Osteoarthritis?*

At the OAI baseline visit, degenerative cruciate ligaments (OR = 2.15; 95%CI = 1.34, 3.45; *p* = 0.002 Table 2), infrapatellar fat pad signal intensity alteration (OR = 1.98; 95%CI = 1.24, 3.15; *p* = 0.004), medial meniscal pathology (OR = 2.14; 95%CI = 1.33, 3.43; p = 0.002), lateral meniscal pathology (OR = 2.36; 95%CI = 1.47, 3.79; *p* = 0.0004), and large effusion-synovitis volume (effusion cutoff > 9.5cm^3^; OR = 2.15; 95%CI = 1.35, 3.43; *p* = 0.001) were more likely to antedate the development of AKOA when compared to those that did not develop AKOA.

**Table 2:** Baseline Pre-Radiographic Structural Features Associated with Accelerated Knee Osteoarthritis (OA) Over the Next 4 Years

Structural Feature			Group		
			AKOA (*n* = 113)	No AKOA (Reference, *n* = 241)	Univariate Odds Ratio
			n (%)	n (%)	OR (95% CI)
Semi-Quantitative	Degenerative Cruciate Ligaments	Absence	66 (58%)	181 (75%)	Reference
		Presence	47 (42%)	60 (25%)	2.15 (1.34, 3.45)*
	Degenerative Collateral Ligaments	Absence	88 (78%)	206 (85%)	Reference
		Presence	25 (22%)	35 (15%)	1.67 (0.95, 2.96)
	Degenerative Extensor Mechanism	Absence	64 (57%)	163 (68%)	Reference
		Presence	49 (43%)	78 (32%)	1.60 (1.01, 2.53)
	Degenerative Gastrocnemius Tendon	Absence	64 (57%)	146 (61%)	Reference
		Presence	49 (43%)	95 (39%)	1.18 (0.75, 1.85)
	Infrapatellar Fat Pad Signal Intensity Alteration	Absence	63 (56%)	172 (71%)	Reference
		Presence	50 (44%)	69 (29%)	1.98 (1.24, 3.15)*
	Medial Meniscal Pathology	Absence	35 (31%)	118 (49%)	Reference
		Presence	78 (69%)	123 (51%)	2.14 (1.33, 3.43)*
	Lateral Meniscal Pathology	Absence	64 (57%)	182 (76%)	Reference
		Presence	49 (43%)	59 (24%)	2.36 (1.47, 3.79)*
	Medial Meniscal Extrusion	Absence	90 (80%)	210 (87%)	Reference
		Presence	23 (20%)	31 (13%)	1.73 (0.96, 3.13)
	Lateral Meniscal Extrusion	Absence	109 (96%)	238 (99%)	Reference
		Presence	4 (4%)	3 (1%)	2.91 (0.64, 13.23)
Quantitative	Effusion-Synovitis Volume^	Smallest	63 (56%)	176 (73%)	Reference
		Largest	50 (44%)	65 (27%)	2.15 (1.35, 3.43)*
	Bone Marrow Lesion Volume^	Smallest	78 (69%)	161 (67%)	Reference
		Largest	35 (31%)	80 (33%)	0.90 (0.56, 1.46)
	Cartilage Damage Index^	Largest	79 (70%)	159 (66%)	Reference
		Smallest	34 (30%)	82 (34%)	0.84 (0.52, 1.35)

*Statistically significant: *p* < 0.004. ^Comparing the worst tertile (i.e. largest effusion-synovitis /bone marrow lesion, smallest CDI) to the other 2 tertiles

At 2 years prior to disease onset, the same structural features from the OAI baseline analysis were more common in individuals prior to the development of AKOA when compared to those that did not develop AKOA (Table 3). The effusion cutoff at 2 years prior to disease onset was 9.8cm^3^.

**Table 3:** Pre-Radiographic Structural Features at 2 Years Prior to Disease Onset Associated with Accelerated Knee Osteoarthritis

Structural Feature			Group		
			AKOA (*n* = 90)	No AKOA (Reference, *n* = 158)	Univariate Odds Ratio
			n (%)	n (%)	OR (95% CI)
Semi-Quantitative	Degenerative Cruciate Ligaments	Absence	52 (58%)	120 (76%)	Reference
		Presence	38 (42%)	38 (24%)	2.31 (1.33, 4.02)*
	Degenerative Collateral Ligaments	Absence	69 (77%)	140 (89%)	Reference
		Presence	21 (23%)	18 (11%)	2.37 (1.18, 4.73)*
	Degenerative Extensor Mechanism	Absence	50 (56%)	111 (70%)	Reference
		Presence	40 (44%)	47 (30%)	1.89 (1.10, 3.24)*
	Degenerative Gastrocnemius Tendon	Absence	46 (51%)	95 (60%)	Reference
		Presence	44 (49%)	63 (40%)	1.44 (0.86, 2.43)
	Infrapatellar Fat Pad Signal Intensity Alteration	Absence	45 (50%)	112 (71%)	Reference
		Presence	45 (50%)	46 (29%)	2.44 (1.42, 4.17)*
	Medial Meniscal Pathology	Absence	26 (29%)	85 (54%)	Reference
		Presence	64 (71%)	73 (46%)	2.87 (1.65, 4.98)*
	Lateral Meniscal Pathology	Absence	49 (54%)	121 (77%)	Reference
		Presence	41 (46%)	37 (23%)	2.74 (1.57, 4.76)*
	Medial Meniscal Extrusion	Absence	73 (81%)	142 (90%)	Reference
		Presence	17 (19%)	16 (10%)	2.07 (0.99, 4.33)
	Lateral Meniscal Extrusion	Absence	87 (97%)	157 (99%)	Reference
		Presence	3 (3%)	1 (1%)	5.41 (0.56, 52.84)
Quantitative	Effusion-Synovitis Volume^	Smallest	46 (51%)	120 (76%)	Reference
		Largest	44 (49%)	38 (24%)	3.02 (1.74, 5.24)*
	Bone Marrow Lesion Volume^	Smallest	62 (69%)	105 (66%)	Reference
		Largest	28 (31%)	53 (34%)	0.90 (0.51, 1.56)
	Cartilage Damage Index^	Largest	62 (69%)	103 (65%)	Reference
		Smallest	28 (31%)	55 (35%)	0.85 (0.49, 1.47)

*Statistically significant: *p* < 0.004. ^Comparing the worst tertile (i.e. largest effusion-synovitis /bone marrow lesion, smallest CDI) to the other 2 tertiles

At 1 year prior to disease onset, all significant features from the OAI baseline analysis were more common in individuals prior to the development of AKOA when compared to those that did not develop AKOA (Table 4). Additionally, we found that the presence of medial meniscus extrusion (OR = 3.52; 95%CI = 2.07, 6.00) increased the likelihood of developing AKOA when compared to individuals who did not develop AKOA. The effusion cutoff at 2 years prior to disease onset was 11.9cm^3^.

**Table 4:** Pre-Radiographic Structural Features at 1 Years Prior to Disease Onset Associated with Accelerated Knee Osteoarthritis

Structural Feature			Group		
			AKOA (*n* = 112)	No AKOA (Reference, *n* = 242)	Univariate Odds Ratio
			n (%)	n (%)	OR (95% CI)
Semi-Quantitative	Degenerative Cruciate Ligaments	Absence	64 (57%)	180 (74%)	Reference
		Presence	48 (43%)	62 (26%)	2.18 (1.36, 3.49)*
	Degenerative Collateral Ligaments	Absence	86 (77%)	206 (85%)	Reference
		Presence	26 (23%)	36 (15%)	1.73 (0.99, 3.04)
	Degenerative Extensor Mechanism	Absence	62 (55%)	165 (68%)	Reference
		Presence	50 (45%)	77 (32%)	1.73 (1.09, 2.74)*
	Degenerative Gastrocnemius Tendon	Absence	63 (56%)	140 (58%)	Reference
		Presence	49 (44%)	102 (42%)	1.07 (0.68, 1.68)
	Infrapatellar Fat Pad Signal Intensity Alteration	Absence	53 (47%)	169 (70%)	Reference
		Presence	59 (53%)	73 (30%)	2.58 (1.63, 4.09)*
	Medial Meniscal Pathology	Absence	23 (21%)	115 (48%)	Reference
		Presence	89 (79%)	127 (52%)	3.50 (2.08, 5.91)*
	Lateral Meniscal Pathology	Absence	63 (56%)	180 (74%)	Reference
		Presence	49 (44%)	62 (26%)	2.26 (1.41, 3.62)*
	Medial Meniscal Extrusion	Absence	72 (64%)	209 (86%)	Reference
		Presence	40 (36%)	33 (14%)	3.52 (2.07, 6.00)*
	Lateral Meniscal Extrusion	Absence	103 (92%)	237 (98%)	Reference
		Presence	9 (8%)	5 (2%)	4.14 (1.36, 12.66)
Quantitative	Effusion-Synovitis Volume^	Smallest	46 (41%)	190 (79%)	Reference
		Largest	66 (59%)	52 (21%)	5.24 (3.23, 8.52)*
	Bone Marrow Lesion Volume^	Smallest	69 (62%)	169 (70%)	Reference
		Largest	43 (38%)	73 (30%)	1.44 (0.90, 2.31)
	Cartilage Damage Index^	Largest	73 (65%)	164 (68%)	Reference
		Smallest	39 (35%)	78 (32%)	1.12 (0.70, 1.80)

*Statistically significant: *p* < 0.004. ^Comparing the worst tertile (i.e. largest effusion-synovitis/bone marrow lesion, smallest CDI) to the other 2 tertiles

### Secondary analysis

*Which Combination of Baseline Pre-Radiographic Structural Features Most Associate with the Onset of Accelerated Knee Osteoarthritis?*

At the OAI baseline, the combination of medial meniscal pathology, degenerative cruciate ligaments, greater effusion-synovitis volume, and lateral meniscal pathology provided good discrimination between individuals that would develop AKOA within the next four years and individuals that would not develop AKOA (C-statistic = 0.70).

At 2 years prior to disease onset, the pre-radiographic structural features included in the OAI baseline analysis plus degenerative collateral ligaments provided good discrimination between individuals that would develop AKOA within the next four years and individuals that would not develop AKOA (C-statistic = 0.76).

At 1 year prior to disease onset, the combination of medial meniscal pathology, degenerative cruciate ligaments, greater effusion-synovitis volume, infrapatellar fat pad signal intensity alteration, and medial meniscal extrusion provided good discrimination between individuals that would develop AKOA within the next four years and individuals that would not develop AKOA (C-statistic = 0.77).

The sensitivity analyses in the OAI baseline and the one year prior to disease cohorts that limited the sample size to the 248 individuals (i.e., participants included in the 2 years prior to disease cohort) did not alter the findings of any of the analyses.

## Discussion

In this longitudinal study we found that several structural pathologies preceding the onset of radiographic knee OA increased the risk for the subsequent development of AKOA compared to individuals who did not develop AKOA. At the OAI baseline visit, the presence of degenerative ligaments, effusion-synovitis, and meniscal pathology were identified as pre-radiographic structural features that identified an increased risk of AKOA development over the next four years. Medial meniscal extrusion was additionally associated with AKOA at 1 year before the onset of disease. Thus, these pre-radiographic structural features, especially these more proximate findings, antedate the development of AKOA and may help identify individuals likely to develop AKOA in the near future.

These results were consistent, and not attenuated, even when mutually adjusted for in multivariate models, as we observed that the combination of medial meniscal pathology, degenerative cruciate ligaments, and the greatest tertile of quantitative knee effusion-synovitis volume (> 9.5cm^3^) at OAI baseline were associated with the future development of AKOA. Furthermore, the pre-radiographic structural features that associated with AKOA depended on the proximity of time between the image assessment and the onset of disease. Specifically, at 2 years prior to disease development, lateral meniscal pathology and degenerative collateral ligaments were also associated with AKOA and included in the multivariate model. At the year prior to disease development, infrapatellar fat pad signal intensity alteration and medial meniscal extrusion were associated with AKOA and included in the multivariate model. Therefore, depending on the time to disease onset, different combinations of pre-radiographic structural features may be most indicative of future AKOA development and may help us eventually determine their risk of AKOA over 1, 2 or 4 years.

We consistently observed that regardless of time, the presence of meniscal pathology was associated with future AKOA development. These findings complement previous research where we observed that incident AKOA was often characterized by medial meniscal tears with moderate-severe extrusion or changes in meniscal size [5], as well as other studies that have observed that meniscal pathology [20] and meniscal extrusion [21] were related to knee OA onset. Additionally, individuals with medial meniscal extrusion at the year prior to disease development are approximately 3.5 times more likely to develop AKOA. Meniscal extrusion was not associated with AKOA at any other timepoints. This indicates that medial meniscal extrusion may be a later finding that becomes relevant in the year prior to the onset of advanced-stage disease (KL = 3 or 4). While our analyses limit us from making causal inferences, previous biomechanical studies [22, 23] have observed that medial meniscal pathology results in increased tibiofemoral contact pressure and alterations in knee kinematics which may lead to overloading of the knee joint. Additionally, meniscal pathology and meniscal extrusion are key risk factors for fast cartilage loss [24–26]. Therefore, disruption of the medial meniscus may be related to the rapid decline in joint health which is why there is an association with future AKOA development.

Our results indicate that regardless of time, individuals with cruciate ligament degeneration are more than twice as likely to develop AKOA. Despite the apparent importance of cruciate ligament degeneration, the main semi-quantitative whole joint scoring systems only assess for acute tear and do not provide an indicator of cruciate ligament degeneration [27, 28], even though previous findings have observed an association between degenerative cruciate ligaments and symptomatic KOA [29]. The main function of the cruciate ligaments are to facilitate rotational and translational stability to the knee joint [30], and degenerative cruciate ligaments present with altered fiber arrangement and collagen composition [31, 32]. Additionally, individuals with cruciate ligament degeneration present with greater severity of cartilage damage, bone marrow lesions, subchondral cysts, and lateral meniscus pathology when compared to individuals with normal cruciate ligaments [33]. While we are unable to make definitive claims based on our results, cruciate ligament degeneration could be early evidence of a maladaptation to loading or due to aberrant joint loading created by impaired ligamentous function that increases knee instability or laxity [34]. Future studies are needed to confirm whether the presence of knee instability increases the risk for incident AKOA. If instability is present, then it may explain the increased meniscal damage and large effusion-synovitis commonly observed among knees that develop AKOA [5].

Knee effusion-synovitis volume may be the pre-radiographic structural feature most strongly associated with future AKOA development. At 2 years prior to disease development, individuals with effusion-synovitis greater than 11.9 cm^3^ are about 3 times more likely to develop AKOA, with this likelihood increasing to ~ 5.2 times in the year prior to the onset of advanced-stage disease. Effusion-synovitis has previously been observed to precede radiographic knee OA, and is theorized to be a consequence of early, underlying damage occurring within the knee [20, 35]. Therefore, all of the pathologic and degenerative tissues in the joint may contribute to greater knee effusion-synovitis. Furthermore, the effusion-synovitis may be a secondary sign of maladaptation to loading that is stressing other tissues in the joint. This study offers new knowledge indicating that individuals in the greatest tertile effusion-synovitis prior to OA onset are more likely to develop AKOA when compared to individuals with less effusion-synovitis. Individuals with AKOA have greater pain than those with typical knee OA [2] and this may be partially attributable to their large effusion-synovitis, which is associated with increased pain [36, 37]. Therefore, the association between AKOA and increased pain may be mediated by greater knee effusion-synovitis. However, future investigations are needed to determine the specific mechanisms leading to increased pain and effusion-synovitis in individuals with knee OA.

This study provides a critical initial step in determining which pre-radiographic structural features may serve as future prognostic imaging markers of AKOA; however, there are some limitations that must be discussed. Our analyses are not able to provide evidence of specific causal pathways between the identified pre-radiographic structural features and AKOA development, but only that the presence of these features precedes the eventual AKOA development. Future studies are needed to confirm that these structural features are prognostic and mechanistically involved in the development of AKOA. This study indicates which individual pre-radiographic structural features may increase the risk of AKOA development, however, we are unable to confirm how each pathologic finding is dependent on another. Therefore, future studies are needed to determine if each structural feature is a different pathway to the same outcome (i.e. AKOA), if the features are various manifestations due to the same underlying process, or if there are particular combinations of features leading to AKOA. We assessed knee effusion-synovitis using non-contrast enhanced MR images even though contrast-enhanced MR images are considered the gold standard. Due to the possible complications, increased price, lack of clinical feasibility in a pre-radiographic population the non-contrast MR was selected for the OAI protocol [10]. However, even when using the non-contrast MR imaging, we observed significant associations between knee effusion-synovitis and AKOA development. Meniscal tears of different morphologies were collapsed into the same variable of meniscal pathology due to small sample sizes of the individual types of tears. Since different meniscal tears have different biomechanical significance to the knee [38], future studies should attempt to individually determine the significance of specific meniscal tears. Two musculoskeletal radiologists performed the semi-quantitative structural readings with an uneven distribution of cases (RW = 225, JM = 120); however, our readers demonstrated good agreement that is similar to previous semi-quantitative readings [39].

## Conclusions

In conclusion, this study indicates specific early structural features (e.g. degenerative ligaments, effusion/synovitis, and meniscal) that may be evidence of early maladaptation to loading that precedes the onset of AKOA. These findings should be considered as potential prognostic biomarkers that warrant further study.

## Abbreviations

AKOA: Accelerated knee osteoarthritis
BML: Bone marrow lesions
CDI: Cartilage damage index
CI: Confidence intervals
ICC: Intraclass correlation coefficients
KL: Kellgren-Lawrence
MR: Magnetic resonance
OA: Osteoarthritis
OAI: Osteoarthritis Initiative
OR: Odds ratio
